# A neurobiological perspective on social influence: Serotonin and social adaptation

**DOI:** 10.1111/jnc.15607

**Published:** 2022-03-31

**Authors:** Patricia Duerler, Franz X. Vollenweider, Katrin H. Preller

**Affiliations:** ^1^ Neuropsychopharmacology and Brain Imaging, Department of Psychiatry, Psychotherapy and Psychosomatics University Hospital for Psychiatry Zurich Zurich Switzerland

## Abstract

Humans are inherently social beings. Being suggestible to each other's expectations enables pro‐social skills that are crucial for social learning and adaptation. Despite their high relevance for psychiatry, the neurobiological mechanisms underlying social adaptation are still not well understood. This review, therefore, provides a conceptual framework covering various distinct mechanisms underlying social adaptation and explores the neuropharmacology — in particular the role of the serotonin (5‐HT) system — in modulating these mechanisms. This article reviews empirical results on social influence processing and reconciles them with recent findings from psychedelic research on social processing to elucidate neurobiological and neuropharmacological underpinnings of social adaptation. Various computational, neurobiological, and neurochemical processes are involved in distinct mechanisms underlying social adaptation such as the multisensory process of social information integration that is crucial for the forming of self‐representation and representations of social norms. This is again associated with self‐ and other‐perception during social interactions as well as value‐based decision‐making that guides our behavior in daily interactions. We highlight the critical role of 5‐HT in these processes and suggest that 5‐HT can facilitate social learning and may represent an important target for treating psychiatric disorders characterized by impairments in social functioning. This framework also has important implications for psychedelic‐assisted therapy as well as for the development of novel treatment approaches and future research directions.

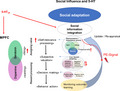

## Graphical abstract

Our social environment has a constant influence on our behaviour. Nevertheless, our understanding of the neurobiopharmacological underpinnings of social influence processing is still limited. This review provides a conceptual framework covering distinct mechanisms underlying social adaptation. It specifically elucidates the fundamental role of serotonin suggested to facilitate social learning by modulating social information integration. Recent pharmacological challenge studies with psychedelics suggest that in particular the serotonin 2A receptor plays a crucial role in the forming of self‐representations and social norms. Therefore, this framework has important implications for psychedelic‐assisted therapy as well as the development of novel treatment approaches in psychiatry.

**Abbreviations**
DA: dopamine
dmPFC: dorsomedial prefrontal cortex
dACC: dorsal anterior cingulate cortex
LSD: lysergic acid diethylamide
mPFC: medial prefrontal cortex
NAcc: nucleus accumbens
PE: prediction error
Psi: psilocybin
5‐HT: 5‐hydroxytryptamine, Serotonin
RN: raphe nuclei
RPE: reward prediction error
SSRI: selective serotonin reuptake inhibitor
vmPFC: ventromedial prefrontal cortex
VS: ventral striatum
VTA: ventral tegmental area

## 1 INTRODUCTION

Humans are inherently social beings. Most of an individual's decisions and actions are made in response to other individuals (Cascio et al., 2015; Challis & Berton, 2015). Social influence processing is a fundamental neurobiological and psychological mechanism as our brain structure and our behavior are constantly shaped by our social experiences and social learning (Henrich & McElreath, 2003). Importantly, we not only learn from direct social experiences or observations but in addition, our behavior is highly susceptible to the expectations of others, that is, social norms (Cialdini & Goldstein, 2004). Given the extensive impact of social influence on our behavior, processes of social adaptation are of great interest for diverse fields ranging from psychology, medicine, sociology, political science, and marketing to basic everyday activities such as communicating with each other in real life or virtually.

Neurobiological mechanisms underlying social influence processing and social adaptation are rooted in our value and reward system and represent fundamental drivers of our motivation and behavior (Campbell‐Meiklejohn et al., 2010; Klucharev et al., 2009; Zaki et al., 2011). In other words, inferring value from each other and the processing of social reward cues guide our behavior (O'Doherty et al., 2017; Scholz et al., 2019). Social influence processing, therefore, involves an interplay of our motivational, affective, and cognitive systems which are again shaped by interaction with the social environment and formed by our social experiences (Challis & Berton, 2015; Edelson et al., 2011; Hughes et al., 2018). Any disturbance in one of these systems can affect social functioning. Impairments in social cognition and interpersonal difficulties are one of the most prominent characteristics and most disabling aspects of various psychiatric disorders (Challis & Berton, 2015; Redcay & Schilbach, 2019; Schilbach, 2016). Importantly, the quality and quantity of interpersonal relationships not only has an impact on mental health but also on physical well‐being as it is directly linked to morbidity and mortality (Holt‐Lunstad et al., 2010).

Social influence has been extensively studied in several domains with a particularly long history in social sciences. Recently, modern methods and techniques in social neuroscience, such as computational accounts or brain imaging techniques, are increasingly used and contribute to a better understanding of the neurobiological mechanisms involved in social influence processing (Campbell‐Meiklejohn et al., 2010; Constant et al., 2019; Klucharev et al., 2009; Li et al., 2020; Lockwood & Klein‐Flügge, 2020; Zaki et al., 2011). However, some major knowledge gaps remain. In particular, the neuropharmacology of social adaptation processes has received little attention so far despite its high relevance for psychiatry, given that therapy for mental illnesses always involves social elements (Norcross & Wampold, 2011). While certain neurotransmitters and neuropeptides, such as dopamine (DA) and oxytocin, have been shown to modulate processes of social adaptation (Campbell‐Meiklejohn et al., 2012; Ebert & Brüne, 2018; Liu et al., 2013; Stallen et al., 2012; Zebrowitz et al., 2018), the 5‐HT system is strongly implicated in social cognition (Lesch, 2005; Zhang & Stackman, 2015). Yet its role in social influence processing is not understood well. Serotonergic psychedelics, such as lysergic acid diethylamide (LSD) or psilocybin (Psi), exert their psychological effects primarily via 5‐HT_2A_ receptor activation (Halberstadt & Geyer, 2013; Vollenweider et al., 1998; Vollenweider & Preller, 2020) and modulate mechanisms involved in emotion (Dolder et al., 2016; Kometer et al., 2012; Kraehenmann et al., 2015; Schmidt et al., 2013) and self‐relevance processing (Duerler et al., 2021; Kraehenmann et al., 2015; Preller et al., 2017; Smigielski et al., 2020; Vollenweider & Preller, 2020), therefore offering a unique opportunity to investigate the neuropharmacological mechanisms of social adaptation. Importantly, they have been shown to alter social cognition and suggestibility as well as brain activity and connectivity of the medial prefrontal cortex (mPFC), a region that is strongly involved in the processing of social information (Duerler et al., 2020; Pokorny et al., 2017; Preller et al., 2016; Preller, Schilbach, et al., 2018).

This review summarizes current findings on the computational and neurochemical underpinnings of social influence processing and highlights the role of the 5‐HT system in these processes. We reconcile these results with findings from psychedelic research on social cognition to reveal novel insights about the role of the 5‐HT_2A_ system in social influence processing. Taking into account computational frameworks such as “predictive coding” and neuroimaging results help to provide a more comprehensive interpretation of the underlying neurobiological mechanisms, in particularly, the role of the 5‐HT system involved in distinct components of social adaptation such as social information integration processing as well as value‐based decision‐making.

In the first part, we provide a theoretical background and review literature on basic mechanisms of social adaptation and learning. Based on this theoretical framework, we then continue to review more recent findings from social neuroscience and neuropharmacological studies for a clearer understanding of the involved neuronal networks and underlying neuropharmacological mechanisms of important distinct components in social adaptation processing. Furthermore, results from psychedelic research—mainly based on studies conducted with psilocybin and LSD investigating the role of the 5‐HT_2A_ receptor—not only contribute to a more differentiated understanding of social influence processing but further extent our knowledge by demonstrating how central processes of social adaptation are interrelated, such as the process of social information integration with the forming of a coherent self‐experience during social interactions. Lastly, implications for psychedelic‐assisted therapy and novel therapeutics are discussed.

## 2 MECHANISMS OF SOCIAL ADAPTATION AND LEARNING

From an evolutionary perspective, the ability to adapt is central for survival across all species and is achieved through learning skills (van Vugt & Kameda, 2014). In humans, in particular, the ability to learn from each other through social interactions facilitated the achievement of adaptation skills and formed the plasticity of the human social brain (Moore, 2004; Richerson & Boyd, 2020; Tomasello, 2020). Being influenceable is the foundation of social learning which is crucial for survival, successful social interactions, as well as for adaptive value‐based decision‐making (Joiner et al., 2017). Furthermore, the processing and integration of social information have been highlighted as vital for the forming of one's own self‐representation, beliefs, and self‐awareness (Collins, 2001; Festinger, 1954; Tsakiris, 2017).

### 2.1 The concept of social alignment

The intrinsic motivation of people to be socially connected and conform is embedded in the concept of social alignment. Social alignment is critical for communicating and learning from each other and leads to feelings of belongingness (Cialdini & Goldstein, 2004; Gallotti et al., 2017; Shamay‐Tsoory et al., 2019). Mechanisms underlying social alignment enable social connection and participation by providing a shared reality and reducing uncertain social environments (Gallotti et al., 2017) and more generally also form the basis of adaptation and learning (Cialdini & Goldstein, 2004). Social alignment involves multi‐level, dynamic, and interactive mechanisms that underlie the sharing and inferring of people's mental representations in all kinds of social interactions (Dale et al., 2014; Gallotti et al., 2017).

### 2.2 Social conformity as part of social alignment

Social conformity can be conceptualized as a form of cognitive alignment with a group and has been defined as an act of changing one's attitudes, beliefs, or behaviors to match social norms that are implicitly or explicitly shared by a group of individuals (Cialdini & Goldstein, 2004; Shamay‐Tsoory et al., 2019). In the long history of social influence research, various reasons for social conformity have been suggested (Wood, 2000). In general, two main motivational drivers rooted in our brain's reward and value system determine our behavior and direct our decision‐making: 1) to approach rewards (e.g., receive social rewards) and 2) to avoid punishments (e.g., avoid social sanctions) (Constant et al., 2019; Lockwood & Klein‐Flügge, 2020; Ruff & Fehr, 2014; Williams et al., 2020). In general, humans have a deep need to be socially connected, being in line with others, and not being rejected (Klucharev et al., 2009; Lieberman, 2007) as social relationships enhance communication and serve self‐relevant advantages that are important for survival (Baumeister & Leary, 1995). Furthermore, humans share an intrinsic preference to be in line with others even when no explicit gain or social sanction is expected (inconsequential conformity) and disagreeing with others feels discomforting (Klucharev et al., 2009; Mistry & Liljeholm, 2019). It is suggested that individuals find intrinsic value in sharing information. Sharing increases activity in neural regions involved in positive valuation and self‐related processing (Baek et al., 2017). Social conformity therefore can be conceptualized as a form of reinforced social learning (Bartra et al., 2013; Falk & Scholz, 2018; Izuma, 2013; Klucharev et al., 2009) and being in line with others feels rewarding and directly affects the neuronal value system (Zaki et al., 2011). Conforming therefore leads to social rewards and not conforming can lead to social punishment in form of social rejection (Falk et al., 2012).

### 2.3 Embodied perspective on social learning

An embodied perspective of social cognition suggests that the starting point of social learning and adaptation is the multisensory comparison of the self with others through social alignment (Tajadura‐Jiménez et al., 2012). Activated biological systems that promote mimicry and synchronization of body and mind allow us to understand the experiences, thoughts, and emotions of others, thus increase social awareness (Cacioppo & Cacioppo, 2012; Falk & Scholz, 2018). Furthermore, by finding similarities and associations as well as differences via the self‐other comparison, self/other boundaries and a sense of self‐identity are enabled (Tajadura‐Jiménez et al., 2012; Tsakiris, 2017). Perceived similarities between self and other may activate self‐associations (Tsakiris, 2017). A stable self‐representation is central in processes of social alignment as it serves as a reference point and enhances the binding of features between different stages of processing during the multisensory self‐other comparisons that is critical for social learning and adaptation (Sui & Humphreys, 2017). Flexible responding to changing social environments requires a stable self‐representation (that is continuously formed and updated through daily social experiences) to provide the necessary balance between adaptability and stability. Social alignment, therefore, relies on both 1) a balanced plasticity to ensure assimilation of changes and updating of self‐representation as well as 2) a sense of continuity one's self‐concept over time (Tajadura‐Jiménez et al., 2012; Tsakiris, 2017).

In contrast, a too rigid or narrow self‐representation (e.g., in depressed patients who often show reduced plasticity of self‐representations [Liu et al., 2017; Northoff, 2014]) or, on the other hand, a too malleable and unstable self (e.g., in schizophrenic patients who show aberrances in plasticity of self‐representation, [Mow et al., 2020; Stephan et al., 2009]) induces an imbalance between stability and adaptability of self‐representation to one side. Therefore both can be maladaptive and often involve aberrances or biases in the processing of social information integration that in turn can hinder the process of forming and “accurate” update of self‐representation (as well as other representation or beliefs) (Sui et al., 2012; Tajadura‐Jiménez et al., 2012; Tsakiris, 2017).

These imbalances in plasticity are often associated with alterations in self‐relevance processing that affect social information integration to the self and suggested to contribute to alterations of self‐other boundaries and self‐perception that are observed in many psychiatric disorders (Markus & Wurf, 1987; Northoff et al., 2006; Northoff, 2014; Feinberg, 2011; Stephan et al., 2009; Mow et al., 2020; Liu et al., 2017; (Sui & Humphreys, 2017). For example, depressed patients often show reduced plasticity of self‐representations associated with altered self‐relevance processing including a narrow, heightened self‐focus with rigid ruminative thinking style, negatively biased self‐associations while at the same time reduced associations of positive emotions with the self (Liu et al., 2017; Northoff, 2014; Renner et al., 2015). This form of altered plasticity of self‐representation can lead to rigid, negatively biased, dysfunctional self‐views that negatively bias social cognition, potentially reinforces maladaptive mechanisms, and can lead to difficulties in social interactions (Clark & Beck, 2010; Mow et al., 2020; Taylor Tavares et al., 2008). Therefore, the balance between bottom‐up and top‐down processes during social information processing is crucial for a sufficiently balanced plasticity of self‐representation, whereas imbalances can disrupt processes of alignment, therefore hinder social learning and adaptation (Challis & Berton, 2015; Stephan et al., 2009; Corlett et al., 2007). Additionally, interoceptive awareness is suggested to play a central role in the ability to distinguish self from other and for social cognition in general (Critchley & Garfinkel, 2015; Palmer & Tsakiris, 2018). Furthermore, these impairments can alter sensitivity for social rewards. This in turn affects value‐based decision‐making that guides our behavior and potentially reinforces maladaptive mechanisms in social interactions (Duranton & Gaunet, 2016; Shamay‐Tsoory et al., 2019; Tsakiris, 2017). For example, pathological gamblers often present with a shift in self‐relevance of personally important habits to gambling as the only personal relevant activity (Greck et al., 2010).

### 2.4 The role of social norms—shared expectations, stability, and predictability

A key element to better understand processes of social influence, particularly social conformity behavior, is the concept of social norms as they help to understand and predict people's behavior in daily interactions (Yomogida et al., 2017). Social norms, defined as expectations about (social) behavior, are unwritten, socially negotiated, and enforced rules that contain information about generally accepted standards about beliefs and expectations about which behavior is appropriate in a certain social environment (Chung & Rimal, 2016; Rimal & Lapinski, 2015). Thus conforming to norms fulfills an essential regulatory function that maintains collective order and enables coordination within a society (Cialdini & Trost, 1998; Ehrlich & Levin, 2005; Rimal & Lapinski, 2015). Social norms are not fixed but dynamically formed within a society, for example, in social interactions, and understood through communication processes (Rimal & Lapinski, 2015). Social norms can provide a convenient decision‐making heuristic for our daily decisions and choices of actions (Cialdini, 2008; Constant et al., 2019; Lapinski & Rimal, 2005).

### 2.5 The social predictive brain

The integration of social information and stimuli is driven by 1) bottom‐up sensory input and 2) cognitive top‐down influences such as social knowledge, expectations, emotional states, past experiences, and contextual factors (Otten et al., 2017; Sonderfeld et al., 2020). Top‐down influence can have a pervasive effect on social cognition and shape how we perceive and interpret the world around us (Barrett & Bar, 2009; Otten et al., 2017; Sonderfeld et al., 2020). Not only social norms per se guide our value‐based decision‐making. Rather, our mental representation of these norms, that is, our expectations, directed attention toward valenced social cues, are important contributors, thereby affecting how we integrate and learn social information, and influencing the value‐based decision process for behavioral action.

Additionally, our ability to infer other people's mental states shapes what we believe others expect (Campbell‐Meiklejohn et al., 2017; Schaafsma et al., 2015; Theriault et al., 2020). Thus, the way we perceive and integrate social information as well as the ability to update it according to our mental representations are critical factors for subsequent decision‐making and choice of behavior as well as for social learning capability in general. To infer what people think and expect in a certain social environment, humans use prior experiences as well as social cues (such as facial expressions, auditory cues, body motions) or other sensory information (Pegado et al., 2018; Williams et al., 2020). Theriault et al. (Theriault et al., 2020) suggested that the motivation to conform to social norms is founded in the predictive nature of our brain and its benefits of maintaining a predictable social environment (Friston, 2010; Theriault et al., 2020). From a predictive coding perspective, the brain uses a predictive model to regulate the body and its interactions with the environment (Friston, 2005; Sterling, 2012). By predictive processing, the brain constantly generates predictions based on previous information (top‐down signals) to anticipate sensory information. When there is a mismatch between the predicted and actual incoming sensory information (bottom‐up signals), the unpredicted signals are called a prediction error signal (PE signal). This theoretical model suggests that the brain minimizes errors and makes neural activity metabolically efficient. To maintain metabolic efficiency, organisms must be able to learn. Therefore, the PE signal is minimized by updating the brain's internal model (Friston, 2010). Unpredictable environments or uncertainty increase metabolic costs. In social environments, these costs can be reduced by conforming to each other's expectations (Theriault et al., 2020). Even if processes of conforming to each other's expectations (social norms) and the ongoing update of self‐representations (social learning) initially involve metabolic costs, these costs can be conceptualized as an investment of the brain to reduce costs and uncertainty in future social situations (Theriault et al., 2020). Therefore, conforming not only helps individuals to be adaptive but also regulates predictability in a social environment by making us predictable to each other (Constant et al., 2019).

It is suggested that the multisensory processes of social alignment help the brain to minimize PEs during social interactions (Bolis & Schilbach, 2017) and that the same principles of predictive coding operate across all levels of social alignment to maximize connectedness and rewards (Shamay‐Tsoory et al., 2019). This regulation of predictability and the reduction of uncertainty also contributes to a shared social reality that creates the necessary conditions for social interactions, thus providing the foundation on which society can be built (Theriault et al., 2020).

Any disruption in the multisensory processing system during social alignment can potentially lead to aberrant PE signaling that hinders social learning and thereby the forming of representations (stable self‐representation/expectations of others) and in the long term may impact social interactions (Apps & Tsakiris, 2014; Corlett et al., 2009; Fletcher & Frith, 2009). Consequently, the adaptive benefits from conforming to others expectations as well as the need for belonging may not be achieved through social interactions (Corlett et al., 2009). The failure to learn from norms or adapt might send wrong signals via inappropriate behavior (Hertz, 2021) that may lead to frustration and loneliness.

### 2.6 Maladaptive forms of social influence

Negative forms of social influence often involve obedience to an ideological doctrine or authority (Haney et al., 1973; Milgram, 1968) and are characterized by maladaptive processes that do not serve the adaptive beneficial function of norms achieved through social learning. Maladaptive norms do not contain “collective wisdom” that reflects the knowledge of many and emerges from shared social interactions and social learning between many individuals (Bettenhausen & Murnighan, 1985). Maladaptive norms rather contain a rigid, predetermined, and unchangeable dogma based on an ideological doctrine or misinformation that are forced and not dynamically shaped through exchange of knowledge (Kendal et al., 2018; Zmigrod, 2020). Conforming to social norms and our often automatically performed heuristics (shortcuts of behavior that are learned to be valuable for expected rewards) can also be exploited for persuasive or manipulating purposes (such as sales or marketing strategies) (Cialdini et al., 1975). This useful human characteristic to be suggestible—fundamental for social learning and adaptation abilities and involving our reward‐ and value system—is also our most vulnerable spot to be exploited for persuasion or manipulation attempts.

## 3 INFORMATION INTEGRATION AND VALUE‐BASED DECISION‐MAKING—SELF‐RELEVANCE AND SUBJECTIVE VALUATION

Processes of social influence can be divided into distinct components, each with potentially different underlying neurobiological mechanisms that are modulated differently by internal and external factors (Ambrase et al., 2020). The depth of social information processing and integration depends on how relevant the information is to the self (Johnson & Eagly, 1989). Self‐relevance directly influences the subjective computation of value that signals expected reward or punishment and guides decision of behavior (Ambrase et al., 2020; Falk & Scholz, 2018). During value‐based decision‐making, choices of action are made on the basis of reward expectations by assigning subjective value to the options available (Ambrase et al., 2020). The brain's reward system is suggested to involve three dissociable neurobiological and psychological processes: liking (hedonic impact of rewarding stimuli), wanting (incentive salience or the approach to reward‐related stimuli), and learning (Berridge et al., 2009).

### 3.1 Self‐relevance processing

When individuals process information they first assess the personal relevance of a stimulus (Sui & Humphreys, 2017, 2017). Recent findings suggest an important link between self‐relevance processing and social cognition (Dinulescu et al., 2021). Self‐relevance processing is suggested to be influenced by the self‐concept or schemas in the long‐term memory based on previous experiences (Dinulescu et al., 2021; Markus & Wurf, 1987). Our representations of ourselves and others flexibly guide social behavior (Adolphs, 2014). Furthermore, greater self‐relevance processing has been associated with greater accuracy in inferring mental states of others (Lombardo et al., 2007). Assigning personal value to self‐related content is essential for forming and stabilizing coherent self‐representations and guiding our decision behavior (D'Argembeau, 2013).

Self‐relevance processing is a key modulator of subjective valuation and also plays a critical role for emotion processing and emotion regulation (Herbert et al., 2011). Reasoning and understanding of emotional reactions to certain stimuli as well as identifying the mental states of others are important for an accurate update and re‐appraisal of mental representations in social interactions (social learning) (Lazarus, 1982; Ludwig et al., 2020; Morrison et al., 2019). Neuroimaging research suggests that computations of self‐relevance and value are highly intertwined (Falk & Scholz, 2018).

### 3.2 Subjective value computation (“liking”) during decision‐making

Decisions to conform involve subjective valuation (Falk & Scholz, 2018). Falk and Scholz (2018) highlight two inputs that are especially important for the neural computation of value in information—self‐relevance (self‐related processing) and social relevance (infer others mental states).

Subjective value computation describes an affective or cognitive process which assigns personal subjective value to a target and is assumed to be based on cost‐benefit weighting of their valence (either positive or negative) based on many decision variables (such as risk, uncertainty, temporal delay, effort) and influenced by emotion, cognition and motivation (Basten et al., 2010; Chong et al., 2017). Subjective value is the result of this cost‐benefit weighting and based on self‐related consequences. Subjective value signals expected reward (reward prediction cues) or punishment and guides decision‐making and the direction of action (approach or avoidance behavior). Evidence from human and rodent studies suggest that the interplay of self‐relevance processing and subjective value computation is related to the concept of “liking” during value‐based decision‐making and contributes to the forming of subjectivity in the valuation signals that reflect subjective preferences (Ambrase et al., 2020; Dölen et al., 2013).

### 3.3 Action selection (“wanting”) process

Value guides the decision of the choice of behavior and signals expected reward when positive or expected punishment when negative (Kahnt & Tobler, 2017) and accounts for past experiences to guide future behavior through the processes of reinforcement learning (Falk & Scholz, 2018). Action selection mechanisms are related to the concept of “wanting” and involve choices of motivation and attribution of incentive salience to stimuli: Action selection describes the choice between approach and avoidance behavior (Berridge & Robinson, 1998; Guitart‐Masip et al., 2014). During action selection, “liking” and “wanting” signals are suggested to interact and determine the timing of the action and effort expenditure based on evidence from rodents (Miyazaki et al., 2012).

## 4 NEURONAL NETWORKS INVOLVED IN SOCIAL ADAPTATION

Recent findings in the field of social neuroscience reveal that processes of adaptation implicate frontal brain regions and neural brain networks related to the computation of value (Campbell‐Meiklejohn et al., 2010; Falk & Scholz, 2018; Klucharev et al., 2009; Mason et al., 2009; Wu et al., 2016). Processes of social adaptation and social learning enabled the forming of our self‐representation and metacognitive abilities which in turn improved the integration of information that promoted adaptation and the plasticity of the brain (Collins, 2001). The mPFC is critical for human cognitive abilities such as complex social information processing (Adolphs, 2014; Smaers et al., 2011), introspection and metacognition (Fleming et al., 2010). The mPFC is coordinating decision‐making toward higher‐level goals (Adolphs, 2014) and has been shown to be a central region for social influence processing. It is modulated by dopaminergic, serotonergic, and noradrenergic inputs, which mediate distinct aspects of decision‐making (Homberg, 2012). Furthermore, the mPFC is suggested to be a central hub for information integration of self‐, reward‐, and mentalizing processes during social feedback processing (Korn et al., 2012) and linked to behavior change during exposure to group norms, supposedly because of its role in the assignment of subjective value to the self (Cooper et al., 2015; Cooper et al., 2017).

### 4.1 Brain regions involved during exposure to group norms and behavior change

Several studies pinpoint the mPFC and the ventral striatum (VS) as key regions for value‐based decision‐making and behavior change (Campbell‐Meiklejohn et al., 2012; Cooper et al., 2017; Mason et al., 2009; Wu et al., 2016; Zaki et al., 2011). Specifically, activation of medial frontal areas and deactivation of the VS during exposure to group norms have been linked to behavior change (Klucharev et al., 2009; Schilbach et al., 2013; Wu et al., 2016). Furthermore, the ventromedial prefrontal cortex (vmPFC) has been shown to regulate conformity behavior linked to value estimation (Clithero & Rangel, 2014). It is suggested that the vmPFC supports adaptive social decisions through self‐relevance processing and the computation of value of social stimuli by integrating this multisensory information with the activity of brainstem structures and subcortical limbic structures that control basic aspects of emotions. Findings from human and rodent studies reveal that other regions controlled by the vmPFC are the amygdala, nucleus accumbens (NAcc) as well regions containing DA and 5‐HT producing neurons (Homberg, 2012; Maren & Quirk, 2004; Verharen et al., 2020).

The vmPFC has been suggested to function as a hub for reward processing together with regions such as the dorsomedial PFC (dmPFC) associated with social reward experience (Eisenberger & Cole, 2012). VmPFC and amygdala functional connectivity has also been found to be involved in the regulation of negative affect (Diekhof et al., 2011)

### 4.2 Brain regions involved during exposure to a mismatch with group norms

When exposed to a mismatch between the own and the group opinion, brain regions associated with conflict detection, reinforcement learning, and social cognition are active (Campbell‐Meiklejohn et al., 2010; Cascio et al., 2015; Klucharev et al., 2009; Schilbach et al., 2010; Wu et al., 2016). Mismatch with group norms often evokes activity in the dmPFC and dorsal anterior cingulate cortex (dACC). Furthermore, people who have a stronger conformity tendency show higher activation in the posterior medial frontal cortex and insula (Berns et al., 2010). During violations of expectations, increased activity in the anterior insula was found in both, social and non‐social contexts (Chang & Sanfey, 2013; Li et al., 2020)

### 4.3 Neural representation of norms

Recent studies highlight the role of the vmPFC in mediating, establishing, and retrieving schemas (Bowman & Zeithamova, 2018; Gilboa & Marlatte, 2017). The neural network of the vmPFC‐hippocampal‐posterior cortical interactions has been identified to be involved in schema‐related functions (Gilboa & Marlatte, 2017). The schematic memory has been suggested to be involved in value‐based decision‐making to optimize reinforcement‐driven behaviors in dynamic environments (Euston et al., 2012; Gilboa & Marlatte, 2017; Santoro et al., 2016). Schemas/representations organize prior knowledge (expectations) that can either promote or also potentially bias the process of decision‐making or the integration of new information. Thus, the posterior vmPFC has been suggested to serve a general role in biasing relevant long‐term representations through processes of assessing self‐relevance and the value of new information in the current context (Haynes et al., 2007)

### 4.4 Social versus non‐social reward processing

The current literature suggests that learning and value‐based decision‐making in social and non‐social contexts relies on similar neuronal computational mechanisms. However, in social situations the brain may engage additional processes to deal with the social environment that facilitate socially relevant tasks, such as inferring other people's mental states (Frith & Frith, 2006); Joiner et al., 2017; O'Doherty et al., 2017).

## 5 SEROTONIN AND SOCIAL ADAPTATION

### 5.1 General functions of the serotonergic system

The role of the 5‐HT system in social influence processing is still not well understood. However, recent neuropharmacological studies revealed new insights into the role of 5‐HT in important underlying distinct components involved in social adaption processing, such as processing of social information and value‐based decision‐making that are critical in our daily social interactions and guide our behavior.

5‐HT exerts a wide range of physiological and behavioral influences through its action on at least 17 different receptors and has a regulatory function throughout the body on the homeostasis system (Cools et al., 2008; Vahid‐Ansari et al., 2019). Therefore, 5‐HT serves multiple roles in many lower‐ and higher‐level functions including mood, emotion, sleep, appetite, reward and punishment learning, cooperation, decision‐making and further homeostatic mechanisms (Jacobs & Azmitia, 1992; Martinowich & Lu, 2008; Roberts et al., 2020). Evidence from human and rodent studies reveals that the majority of 5‐HT cells are located in the raphe nuclei (RN) of the midbrain with ascending fibers projecting to frontal cortex, striatum, thalamus, amygdala, hypothalamus as well as caudal raphe sub‐regions which are interconnected (Bang et al., 2012; Murphy et al., 1998). Recent evidence revealed two main projection subtypes in global mapping: from ventral RN to anterior cortical regions and from dorsal RN to subcortical regions, both with an opposing effect on anxiety and depression phenotypes (Ren et al., 2018). Additionally, different sub‐regions of RN have been associated with depression and anxiety (Commons, 2016). Rodent studies reveal that 5‐HT neurons often co‐express other neurotransmitters, for example, glutamate and GABA (Hioki et al., 2010).

Depletion or boosting of 5‐HT levels via the administration of selective serotonin reuptake inhibitors (SSRIs) or l‐tryptophan has been shown to induce positive and negative alterations in social perception (Crockett et al., 2013; Kanen et al., 2020; Tse & Bond, 2002). Increasing 5‐HT decreases negative feedback sensitivity and increases reward (positive feedback) sensitivity. Furthermore, it increases cognitive flexibility in decision‐making and is suggested to promote adaptive behavior (Homberg, 2012). Additionally, 5‐HT has been shown to promote prosocial behavior potentially via its impact on the vmPFC (Siever, 1999).

Notably, the neurochemical consequences of tryptophan depletion are complex and still not fully understood (Aquili, 2020). Although there is strong evidence that acute tryptophan depletion lowers 5‐HT levels in the brain, it has also been shown that their effect can vary depending on the dose administered (Yatham et al., 2001). Therefore, the exact effects of tryptophan depletion on 5‐HT activity still need clarification (Crockett et al., 2012).

### 5.2 Value‐based decision‐ making—neurobiological mechanisms

Findings from human and rodent studies suggest that value‐based decision‐making is modulated by several neurotransmitters, such as serotonergic and dopaminergic inputs to the mPFC. The mPFC plays a central role in value‐based decision‐making and has been suggested to be central for updating and learning processes (Verharen et al., 2020). The 5‐HT system is suggested to interact with the DA system during the coding of reward prediction errors (RPEs) as some of the RN neurons are connected with the ventral tegmental area (VTA) and NAcc (Liu et al., 2014). RPEs are relayed to the prefrontal and cingulate cortex via dopaminergic projections from the striatum. Serotonergic signals from the RN to frontal cortical areas encode subjectivity in valuation signals and have mainly been associated with the formation of “liking” (Ambrase et al., 2020; Homberg, 2012; Li et al., 2016).

The balance in 5‐HT and DA signaling has been suggested to be necessary for cognitive flexibility and therefore adaptive behavior and optimal decision‐making (Eppinger et al., 2011). Imbalances or altered 5‐HT functions are associated with maladaptive forms of decision‐making (e.g., substance use disorder) (Volkow et al., 2004) or altered regulation of negative feedback mechanisms—often a core element in many psychiatric disorders (e.g., depression) (Dell'Osso et al., 2016; Roberts et al., 2020).

### 5.3 Serotonin modulates subjective valuation (“liking”) related to the assignment of self‐relevance during decision‐making

The interplay of the processes “assigning subjective value” and “self‐relevance processing” is related to the concept of “liking” during value‐based decision‐making. Liking is suggested to be modulated by 5‐HT via its effect on the vmPFC (Ambrase et al., 2020; D'Argembeau, 2013; Fischer & Ullsperger, 2017; Nakamura et al., 2008; Rilling & Sanfey, 2011; Seymour et al., 2012). Self‐relevance processing, a key modulator of subjective valuation (Falk & Scholz, 2018), has further been linked to emotion processing and emotion regulation (Herbert et al., 2011), whereas the 5‐HT system has also been shown to modulate these processes during decision‐making (Crockett et al., 2008). It is therefore conceivable that the 5‐HT system modulates subjective valuation dependent on its influence on self‐relevance processing.

### 5.4 Serotonin and action selection

Increased serotonergic RN activity has been associated with increased willingness to wait longer for rewards in temporal delay tasks by elevating behavioral control of impulsive reactions (Ludwig et al., 2020; Miyazaki et al., 2018). On the other hand, central 5‐HT depletion in lesioned rats has been associated with a general tendency toward impulsive behavior as they were more likely to select a smaller, immediate reward than a larger, delayed reward. In these lesioned rats, 5‐HT levels were reduced but levels of noradrenaline and DA were not altered (Mobini et al., 2000). Therefore, decreased 5‐HT levels may alter the forming of subjective valuation signals (liking signals) by weakening their impact during the process of value‐based decision‐making toward more impulsive “wanting” signals. Although a large number of studies in humans and rodents found an association between reduced 5‐HT levels and the promotion of impulsive behavior (Crockett et al., 2010; Schweighofer et al., 2008), findings obtained on the effect of manipulating 5‐HT levels on impulsive behavior are not always clear and sometimes contradictory (Denk et al., 2005; Homberg, 2012; Liu et al., 2004). Furthermore, impulsive choice behavior is also modulated by other neurotransmitters, especially the interplay of 5‐HT with DA. To clarify the involvement of specific 5‐HT receptor subtypes on impulsive choice behavior, future studies using receptor‐subtype specific 5‐HT agonists or antagonists need to be conducted.

### 5.5 Serotonin and outcome learning

The 5‐HT system is involved in the updating of value for future actions. When an outcome occurs that is better than expected, the signal is called a positive RPE. If the outcome is worse than expected, it is called a negative RPE (Schultz, 2016). Learning from decision outcomes involves integrating past experiences into the value system as well as the ability to update subjective value and representations (expectations) based on these PE signals (Rangel et al., 2008). 5‐HT codes the magnitude of PEs but does not differentiate between positive and negative PEs (Matias et al., 2017).

### 5.6 Serotonin modulates information integration during decision‐making through its effect on the mPFC

**FIGURE 1:**
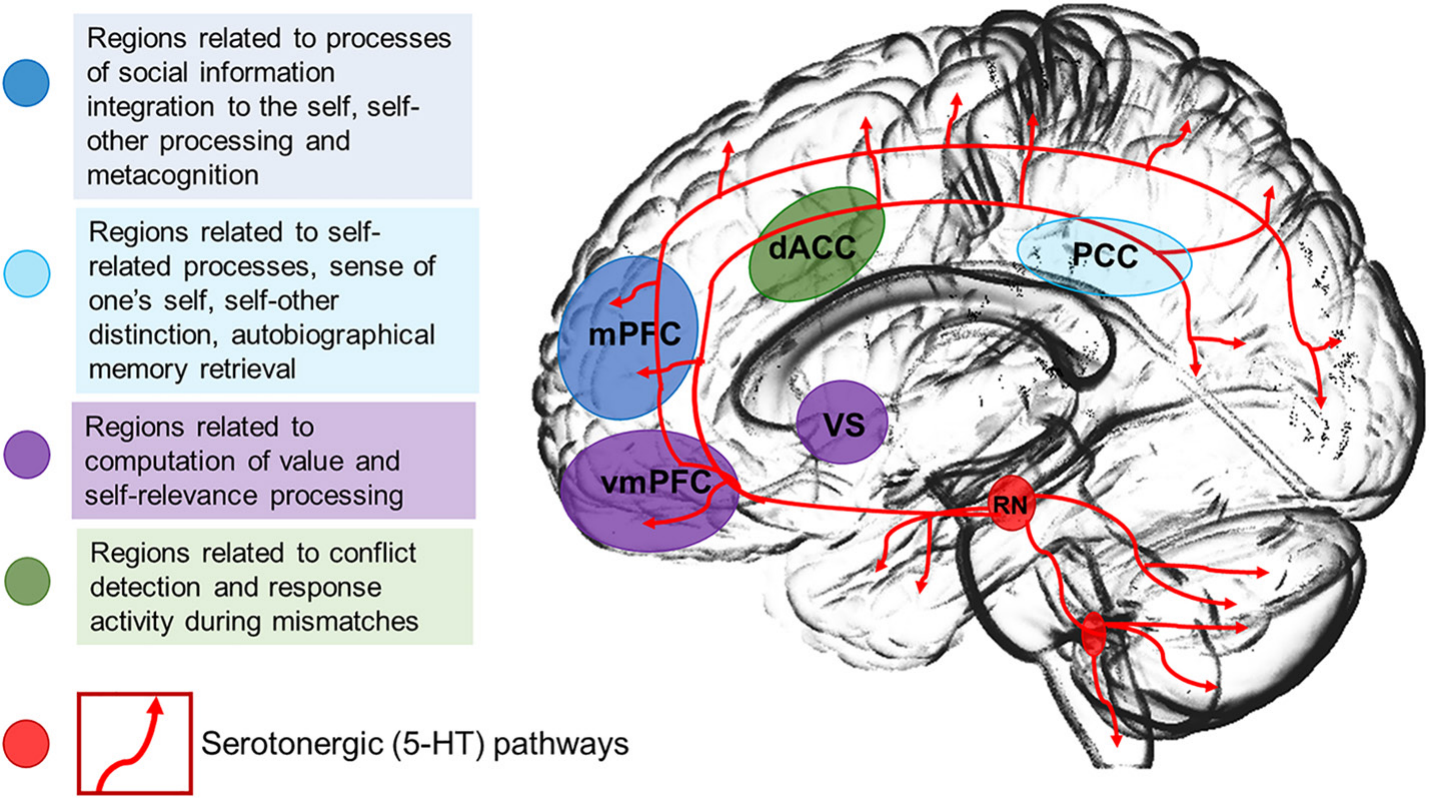
Serotonin and neural networks involved in social influence. Brain regions implicated during behavior change are strongly linked to the brain's reward‐and value system to guide behavior toward adaptive advantages and linked to the process of value‐based decision‐making. 5‐HT has a regulatory function on value‐based decision‐making through modulating the process of information integration via its effect on the mPFC. 5‐HT signals from the RN to frontal cortical areas are suggested to encode subjectivity in valuation signals and modulate the process of assigning value dependent on self‐relevance via its effect on the vmPFC. The 5‐HT system is therefore involved in the updating of value and the forming of representations from new information for future actions. dACC; dorsal anterior cingulate cortex, mPFC, medial prefrontal cortex, vmPFC, ventromedial prefrontal cortex, PCC; posterior cingulate cortex, RN; raphe nuclei, VS; ventral striatum

The mPFC is a key region involved in social conformity (Cooper et al., 2017; Klucharev et al., 2009) and has been shown to be central for the integration of social information in relation to the self via updating the action and outcome relationship. More precisely the mPFC is suggested to assign value to self‐related information during value‐based decision‐making (Figure 1).

Therefore, 5‐HT has as a regulatory function on the value‐based decision process through modulating information integration. This effect may be mediated by the mPFC (Khani & Rainer, 2016). Enhanced 5‐HT signaling facilitates flexible learning, cognitive flexibility, and adaptive decision‐making (Ambrase et al., 2020; Miyazaki et al., 2012). A recent study found that 5‐HT depletion selectively impaired neural representations of reward outcome value, thereby hindering effective comparison between rewards and punishments (Seymour et al., 2012). Depletion of 5‐HT with tryptophan impaired reversal learning in humans, pointing toward its role in cognitive flexibility (Kanen et al., 2020). 5‐HT, therefore, has a central role in the integration of social stimuli and social learning. However, the hypothesis that the 5‐HT system modulates information integration via its effect on the mPFC needs to be tested directly in future studies.

### 5.7 Effects of serotonin on affective processing during information integration

Affective processing also plays an important role in value‐based decision‐making (Krajbich et al., 2009). The activity of areas involved in emotion processing such as the amygdala and insula can therefore also affect value‐based decision‐making and social learning (Rilling & Sanfey, 2011). It is suggested that 5‐HT plays a critical role in emotion regulation during decision‐making (Crockett et al., 2008) by modulating the impact of aversive signals on learning (Cools et al., 2008).

A recent study found that increasing 5‐HT with SSRIs boosts positive affect of subjective value during both, learning of novel, and reconsolidation of previously learned reward associations (Michely et al., 2020). Depleting 5‐HT increased responsiveness to punishment or other aversive signals and reduced behavioral activation (Cools et al., 2008). 5‐HT depletion also gives rise to negative biases in the integration of social information (Harmer, 2008). Another recent study provides evidence that an increase in 5‐HT potentially reduces negative biases in social information integration (both in healthy and depressed individuals) by showing that administration of SSRIs is characterized by changes in responses to expressive faces, specifically increasing neural reactions to positive faces and decreasing reactivity to negative faces (Godlewska & Harmer, 2021).

These findings support the hypothesis that 5‐HT plays a central role in coding the affective value of the environment during decision processes. 5‐HT modulates the processing of social information by increasing the individual's sensitivity to certain social factors that can have either positive or negative consequences depending on the environment (Branchi, 2011). Therefore, 5‐HT facilitates the ability to flexibly adapt to dynamic social environments through plasticity and behavioral control (Matias et al., 2017).

### 5.8 Serotonin promotes self‐regulatory functions

5‐HT modulates the valuation process of rewards (Ludwig et al., 2020) and promotes waiting for temporally delayed rewards if the reward is expected (Miyazaki et al., 2020). 5‐HT thus makes the decision process more adaptive as more information about self‐relevant value can be included in the decision process. Furthermore, metacognitive awareness through self‐reflection or interoception as well as self‐regulation potentially promote adaptive decision‐making as the computation of value relies on reliable insights (Ludwig et al., 2020).

### 5.9 The role of serotonin and dopamine in processing social rewards

A recent study showed that 5‐HT depletion alters social reward prediction signals in the insula, temporal lobe, and PFC and impaired learning from social rewards. DA depletion had a less extensive effect (Frey & McCabe, 2020). Furthermore, lowering 5‐HT levels via tryptophan depletion decreased cooperation behavior (Tse & Bond, 2002) while increasing levels of 5‐HT via SSRI treatment increased cooperation behavior (Wood et al., 2006). Cooperation behavior may seem more valuable as 5‐HT increases the value for long‐term benefits associated with cooperation behavior (Wood et al., 2006).

Although previous findings from human and animal studies strongly implicate the DA system in social conformity (Aquili, 2020; Campbell‐Meiklejohn et al., 2010; Falk et al., 2012; Tobler et al., 2005; Zhao et al., 2016) results are not always consistent on the specific role of DA and its distinction from 5‐HT in the processing of social and non‐social rewards. A recent study found that DA depletion blunts sensitivity to social stimuli, namely to faces varying in trustworthiness (Zebrowitz et al., 2018). Evidence that DA facilitates social conformity behavior is derived from a study where methylphenidate, an indirect DA and noradrenalin agonist, was administered suggesting a mediating role of increased extracellular DA levels in social conformity (Campbell‐Meiklejohn et al., 2012). Furthermore, a recent study demonstrated that the DA receptor 3 gene is associated with individual differences in social conformity behavior (Zhao et al., 2016). Other findings point toward similar functions of DA and 5‐HT in the regulation of executive functions and reward processing (Aquili, 2020; Cools et al., 2011; Rogers, 2011). Despite some differences, DA and 5‐HT are not isolated but rather influence each other during reward processing and decision‐making (Aquili, 2020).

### 5.10 Other neurotransmitters involved in social learning

Other neurotransmitters such as noradrenaline and glutamate have also been shown to modulate social learning in human and rodent studies (Bouret & Sara, 2004; Corlett et al., 2007). Additionally, acetylcholine and noradrenaline have been associated with the signaling of uncertainty (Yu & Dayan, 2005). One prominent neuropeptide that has often been linked to prosocial behavior is oxytocin. It has been shown to promote trust (Nishina et al., 2015; Xu et al., 2019) and enhance empathy (Geng et al., 2018). It augments the rewarding value of social interactions in rats (Ramos et al., 2015). Oxytocin has been shown to alter basic processing of social stimuli depending on the context, for example, salience of interpersonal cues (for a review see (Bartz et al., 2011; Stallen et al., 2012). A recent study found that oxytocin is linked to social feedback learning and subjective valuation by decreasing the value of negative social evaluation. Individuals with high scores in depression tended to devalue positive social evaluation. This was normalized after oxytocin administration (Wang & Ma, 2020). Additionally, growing evidence points toward an important role of oxytocin in promoting interpersonal synchronization (Gebauer et al., 2016; Josef et al., 2019) which is a key component of social alignment.

Furthermore, findings from research in rodents suggest that oxytocin acts as social reinforcement signal by provoking 5‐HT and DA release in the NAcc (Dölen et al., 2013). Oxytocin's interaction with the dopaminergic system is suggested to modulate attentional mechanisms and salience toward social stimuli (Rosenfeld et al., 2011; Shamay‐Tsoory & Abu‐Akel, 2016). Oxytocin, therefore, is suggested to increase salience and the reinforcing value of social cues (Shamay‐Tsoory & Young, 2016). These findings point to a mediating role of oxytocin in social adaptation with similar pro‐social effects as the 5‐HT system, promoting social adaptation and bonding (Wang & Ma, 2020). However, the most relevant differential functions of these neurotransmitters in social adaptation processing as well as their interactions and cascading effects are complex and still not fully understood.

## 6 SEROTONERGIC PSYCHEDELICS AND SOCIAL ADAPTATION

The complexity of the 5‐HT system with its several subreceptors makes it difficult to identify the role of this neurotransmitter system in social adaptation processes. Investigating the role of specific subreceptors may help to create a clearer and more comprehensive understanding of the neuropharmacology of social adaptation. The 5‐HT_2A_ receptor is of particular interest for social adaptation as it has been strongly associated with enhanced neuroplasticity important for adaptation (Azmitia, 2001; Boulougouris et al., 2008; Carhart‐Harris & Nutt, 2017).

The last decade has seen a renaissance of psychedelic research. Especially the application of psychedelics in the clinical field has grown (Bogenschutz & Ross, 2018; Carhart‐Harris et al., 2018; Sessa, 2012). Research with psychedelics has contributed to a better understanding of the role of the 5‐HT system in various functions such as emotion processing (Bernasconi et al., 2015; Kometer et al., 2012; Kraehenmann et al., 2015; Kraehenmann et al., 2016; Schmidt et al., 2013) self‐perception, and social cognition (Hasler et al., 2004; Preller et al., 2016; Preller et al., 2017; Preller, Burt, et al., 2018; Preller, Schilbach, et al., 2018; Preller & Vollenweider, 2019; Studerus et al., 2011). Specifically, psychedelics have been shown to induce acute pro‐social effects and modulate social behavior as well as activity and connectivity of brain areas related to social adaptation (for a review see Preller & Vollenweider, 2019).

Serotonergic psychedelics interact with the 5‐HT system and include semisynthetic ergolines such as LSD and tryptamines such as Psi contained in varies fungi. Both LSD and Psi exert their psychoactive effects primarily via agonistic 5‐HT_2A_ receptor activation (Glennon et al., 1984; Kometer et al., 2013; Preller, Schilbach, et al., 2018; Vollenweider et al., 1998). Findings from rodents revealed that activation of 5‐HT_2A_ receptor by serotonergic psychedelics leads to a glutamate‐dependent increase in pyramidal neurons activity in deep layer (layer V) of the PFC (Aghajanian & Marek, 1997; Puig et al., 2003). While they show high affinity for the 5‐HT_2A_ receptor, they also interact with other serotonergic receptors (e.g., 5‐HT_1A_, 5‐HT_5_, 5‐HT_6_, and 5‐HT_7_) (Nichols, 2004). LSD has in addition agonist activity at DA D2, D1 and a‐adrenergic receptors (Nichols 2016). Administration of the 5‐HT_2A_ antagonist ketanserin has been shown to block Psi and LSD‐induced psychoactive effects (Vollenweider et al., 1998; Kometer et al., 2013; Preller, Burt, et al., 2018). A recent study showed for the first time that ketanserin significantly reduced chronic, intractable visual hallucniation but not auditory hallucination in schizophrenia, suggesting a possible role of the 5‐HT_2A_ in the pathophysiology of visual hallucinations (Sommer et al., 2018). Further evidence also points toward a modulatory influence of the 5‐HT_1A_ receptor in mediating the psychoactive effects of Psi (Carter et al., 2005; Pokorny et al., 2016). In human and rodent studies Psi also showed downstream effects on the DA level in the VS in humans (Puig et al., 2003; Vollenweider, 1999; Vollenweider & Kometer, 2010).

Thus, findings from psychedelic research revealed important insights into the relation between the 5‐HT system and the pathogenesis and treatment of schizophrenia as it helped identify receptor targets for the development of new therapeutic agents (Halberstadt & Geyer, 2013). The second generation of antipsychotics targeting 5‐HT_2A_ antagonistic are under study for schizophrenia and continue to be promising to exert therapeutic effects. In particular, primavanserin and roluperidone are suggested to be the two most promising 5‐HT_2A_ modulators under current investigation (Girgis et al., 2019; Kantrowitz, 2020). Schizophrenia has been associated with social dysfunctions and reduced conformity behavior (Gill, 1963; Marsella, 1975). However, a recent study found no general decline in conformity behavior in antipsychotic medicated patients compared to controls (Simonsen et al., 2019). Suggesting that this “intact” susceptibility to social influence might be a result of antipsychotic treatment and arguing this effect may be mediated by enhanced error signaling in the mPFC through the effect of antipsychotics.

A core characteristic of the psychoactive effects of Psi and LSD is the altering of self‐experience that has been associated with changes in social processing and interaction (Preller et al., 2016; Preller, Schilbach, et al., 2018). Specifically, subjective effects often include profound changes in the sense of self, such as a loosening of self‐other boundaries and the experience of oneness with the surroundings, self‐relevance processing as well as emotion processing—all functions known to be critically involved in processes of social adaptation (Dolder et al., 2016; Preller et al., 2017; Preller & Vollenweider, 2018; Studerus et al., 2011; Vollenweider & Geyer, 2001).

### 6.1 Psychedelics modulate self‐processing and social cognition

LSD has been shown to modulate self‐other processing, specifically decreasing the differentiation between self and other, during a social interaction task via 5‐HT_2A_ receptor stimulation and associated with activity in the mPFC (Preller, Schilbach, et al., 2018). This study points toward a key role of the 5‐HT_2A_ receptor in self‐other processing and demonstrates how self‐processing and social cognition are highly intertwined. Psi has also been shown to alter self‐processing in an EEG/ERP self‐monitoring task designed to assess distinctiveness of self/other sources. Alterations in self‐processing were related to modulations in current source density in brain regions associated with self‐processes such as the ACC and insular cortex and correlated with Psi‐induced feelings of unity (oneness) and changed meaning of percepts (Smigielski et al., 2020).

Additionally, it has been shown that Psi reduces reactions to social exclusion and social pain—and effect that was correlated with alterations in self‐processing and the feeling of oneness (Preller et al., 2016). Another study that highlights the crucial role of the serotonergic 5‐HT_2A_ receptor in self‐relevance processing showed that LSD‐induced increases the attribution of personal meaning to previously meaningless stimuli were also dependent on 5‐HT_2A_ activation (Preller et al., 2017).

### 6.2 Psychedelics increase sensory processing and alter integration of information

Psychedelic‐induced alterations of self‐relevance processing are suggested to be caused by a disruption of activity and connectivity in associative networks including the default mode network (Preller, Burt, et al., 2018). Studies have shown that LSD and Psi acutely increase global functional connectivity in sensory brain areas and thalamic connectivity, while connectivity in associative regions is reduced. This points toward altered integration of information and at the same time increased sensory processing underlying the psychedelic state (Preller et al., 2020; Preller, Burt, et al., 2018). Increased excitatory connections from the thalamus to the posterior cingulate cortex have been suggested to reduce the amount of filtering for ascending PEs (Preller et al., 2019). It has been proposed that a break‐down of top‐down predictions from high‐level association cortices and decomposition into many overly detailed predictions because of 5‐HT_2A_ mediated excitation of layer V pyramidal cells may lead to the psychedelic experiences (van Rooij Sarit Pink‐Hashkes & Kwisthout, 2017). Furthermore, it is suggested that psychedelics may relax the precision weighting of prior beliefs while increasing the bottom‐up flow of information (Carhart‐Harris & Friston, 2019).

The psychedelic‐induced ego‐dissolution characterized as loosening of self‐other boundaries is therefore suggested to relate to a multidimensional experience that results from a disruption in integrating multisensory signals that usually enable the forming of a stable self (Ho et al., 2020; Letheby & Gerrans, 2017). Psychedelics, therefore, produce novel forms of information integration and thus may promote re‐adaptation of maladaptive processes through the re‐structuring of representations.

### 6.3 Psychedelics effects on mood during social information processing

Next to self‐relevance processing, affective processing is also known to play a critical role in social processing. Activation of the 5‐HT_2A_ receptor has been related to mood regulation and emotional face recognition as psychedelics have repeatedly been shown to decrease the processing of negative emotional stimuli (Barrett & Finlay, 2018; Bershad et al., 2016; Dolder et al., 2016; Kometer et al., 2012; Kraehenmann et al., 2015; Schmidt et al., 2013). Emotional empathy—crucial for social interactions and social learning—has been shown to be increased after LSD and Psi administration (Dolder et al., 2016; Holze et al., 2021; Pokorny et al., 2017). Additionally, LSD has been shown to increase oxytocin levels via 5‐HT_2A_ receptor stimulation after a high dose (200 μg) (Holze et al., 2021). Furthermore, psychedelics have acute pro‐social effects such as increases in altruistic behavior (Dolder et al., 2016), openness (Erritzoe et al., 2019), and feelings of connectedness (Carhart‐Harris et al., 2018).

### 6.4 Serotonergic psychedelics and social influence processing

Nevertheless, and despite the high therapeutic relevance of social adaptation, very little research has been conducted to investigate the effect of serotonergic psychedelics on social influence processing. Only three studies have investigated the effect of psychedelics on suggestibility so far (Carhart‐Harris et al., 2015; Duerler et al., 2020; Wießner et al., 2021).

The first study (Carhart‐Harris et al., 2015) showed that LSD (40–80 μg administered intravenously) increases suggestibility on the creative imagination scale (CIS). Similarly, a more recent study using an orally administered dose of LSD (50 μg) also found that LSD increases suggestibility. This was not correlated with other effects such as aberrant salience processing (Wießner et al., 2021). Another study (Duerler et al., 2020) found that LSD (100 μg administered orally) increased adaptation in response to social feedback but only when opinions were not too different from the participant's own. LSD‐induced changes in conformity behavior during social feedback processing were modulated by 5‐HT_2A_ stimulation and associated with increased activity in the mPFC. 5‐HT_2A_ activation was only related to activity changes during social feedback processing rather than the subsequent decision‐making process. This is in line with previous findings showing that risk taking and decision‐making are cognitive domains that are not influenced by stimulation of the 5‐HT_2A_ receptor (Pokorny et al., 2019). It is proposed that LSD‐induced alterations in self‐processing affect the computation of value, which signals expected reward dependent on its self‐relevance in the value‐based decision process for subsequent behavior. Therefore, adaptation to similar opinions may be enhanced and more self‐relevant because of altered self‐processing, that is, loosening self‐other boundaries and feelings of oneness that have previously been shown to alter social interaction and social cognition (Preller et al., 2016; Preller, Schilbach, et al., 2018).

**FIGURE 2:**
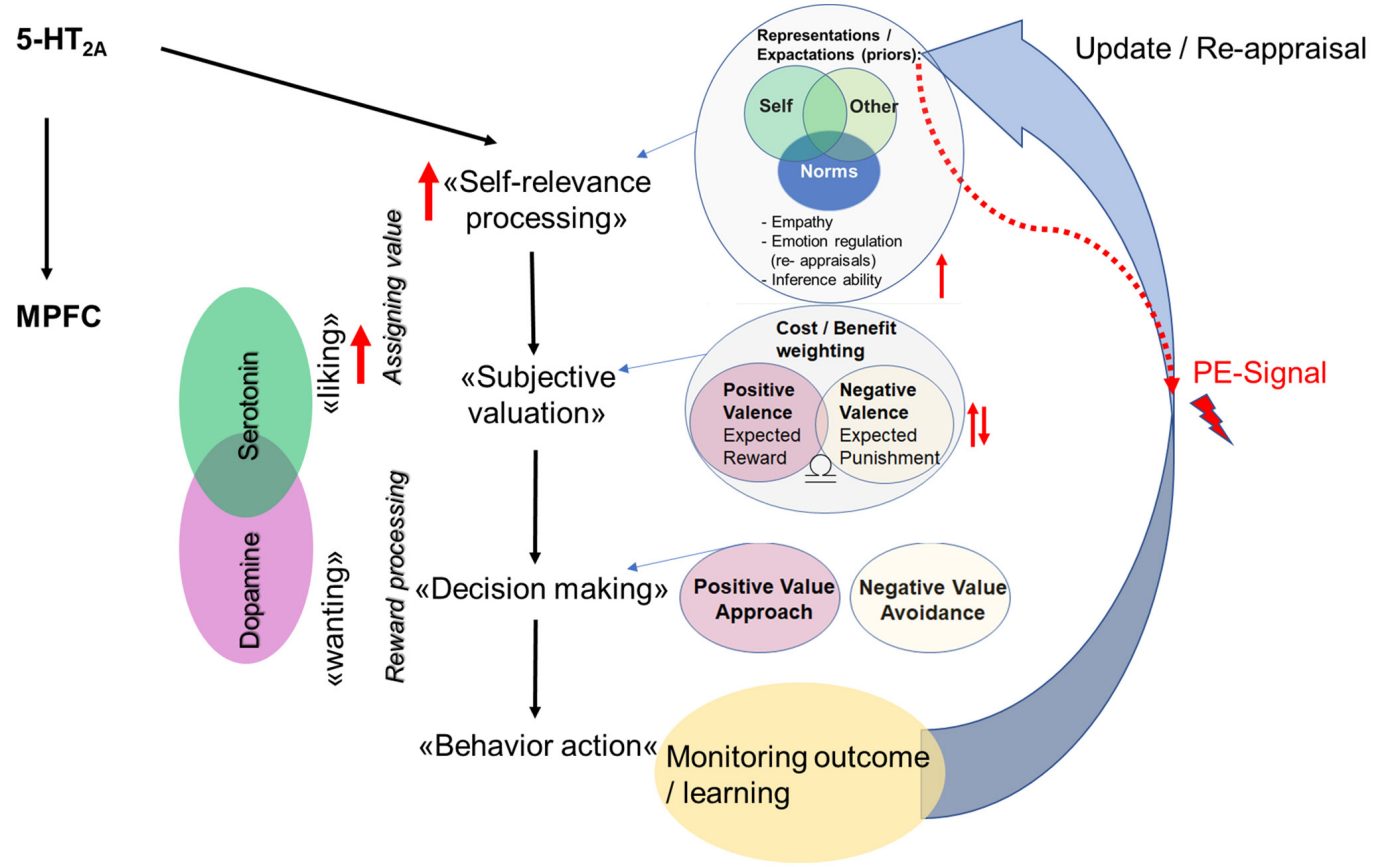
The 5‐H T_2A_ receptor impacts social adaptation processing via the mPFC during the process of information integration. The 5‐HT_2A_ receptor plays a key role in the processing and integration of social information via its effect on the mPFC. It is proposed that stimulation of 5‐HT_2A_ receptor induces alterations in self‐relevance processing that affects computation of value that reflects expected reward signals for subsequent choice of behavior in the process of value‐based decision‐making. The 5‐HT system is therefore suggested to modulate the process of liking during value‐based decision‐making that contributes to the forming of subjectivity in the valuation signals, therefore crucial for learning and updating processes of representations. mPFC, medial prefrontal cortex; PE, prediction error

These findings point toward the important role of the 5‐HT system in value‐based decision‐making and its potential influence on reward processing. Therefore, the 5‐HT system, particularly the 5‐HT_2A_ receptor, plays a key role in the processing and integration of social information via modulation of mPFC function (Figure 2). Psychedelics target the key region for integrating self‐relevant information and thus the basic process for the forming of self‐representation which is crucial for social learning and social adaptation. However, it is noteworthy, that we do not know to what extent an additional modulation of the 5‐HT_1A_ receptor system by psychedelics contributes to the different effects mentioned above (Roberts et al., 2020).

## 7 FUTURE HORIZONS—IMPLICATIONS FOR PSYCHEDELIC‐ASSISTED THERAPY

A growing amount of psychedelic research points toward the potential of these molecules in promoting social adaptation. The subjective experience during the psychedelic state is suggested to play a crucial role in the clinical effects in addition to psychotherapy (Bogenschutz & Ross, 2018). A psychedelic‐induced feeling of connectedness has been suggested to be a key component that can boost treatment efficacy in trials testing Psi for smoking cessation potentially by reinstating social reward processing (Carhart‐Harris et al., 2018; Noorani et al., 2018). Furthermore, subjectively reported increases in interpersonal closeness and positive prosocial effects in healthy participants lasted up to 14 months after the administration of LSD (Schmid & Liechti, 2018).

**FIGURE 3:**
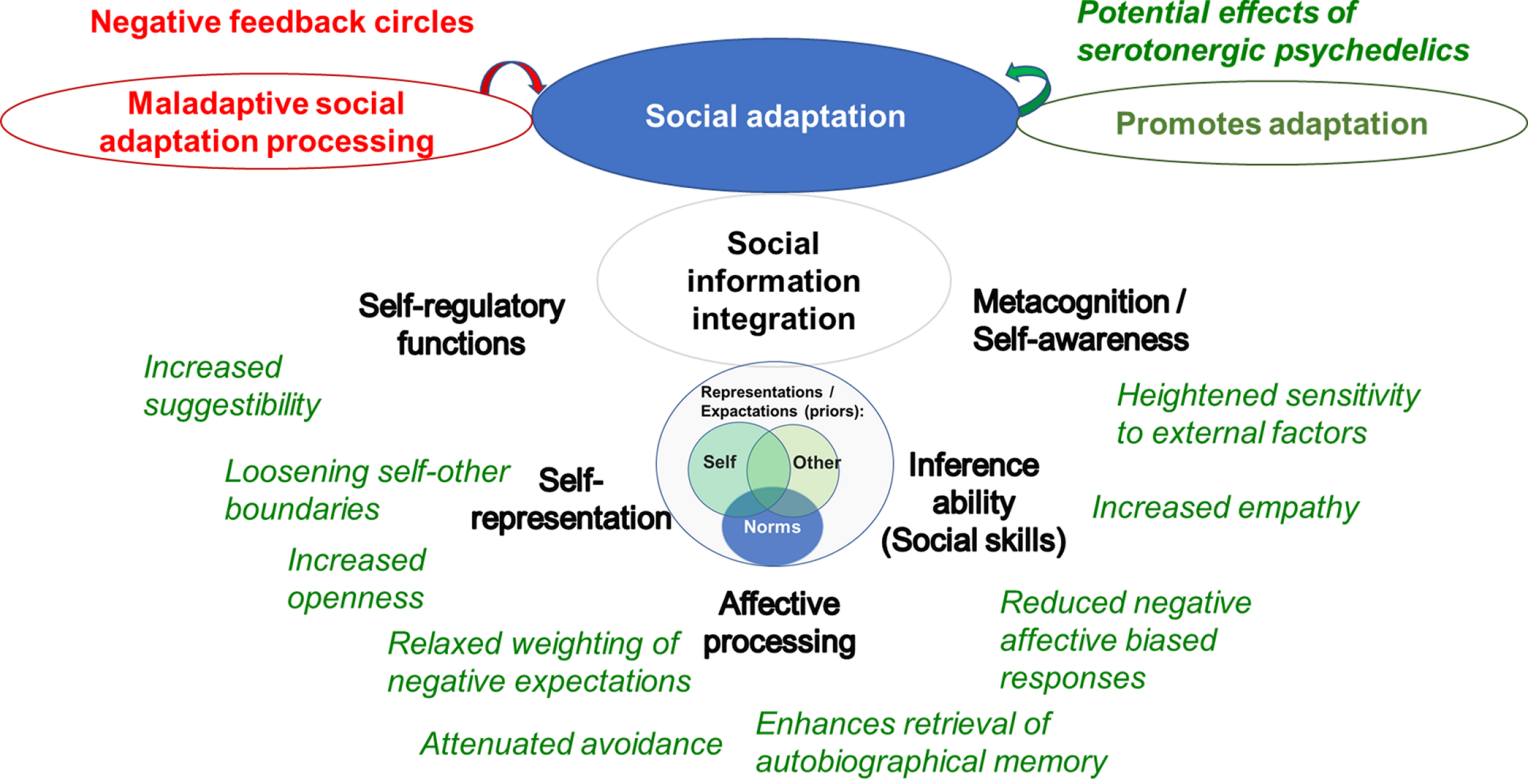
Overview of serotonergic psychedelic‐induced effects that potentially promote social adaptation processing. This figure summarizes findings on the psychedelic‐induced effects that may promote social adaption. The 5‐HT_2A_ receptor has been linked to neuroplastic effects that potentially facilitate social learning and promote social adaptation. Therefore, targeting these processes in psychedelic‐assisted treatment approaches could help a variety of psychiatric disorders by fostering novel forms of information integration of perceiving self and others that potentially re‐structure maladaptive constraints of representations or maladaptive decision‐making

Social adaptation, 5‐HT signaling, and the social environment are highly intertwined (Branchi, 2011). This has important implications for the therapeutic administration of psychedelics. Leveraging the psychedelic‐induced promotion of social functioning and social learning can contribute to therapeutic success (for an overview see Figure 3). Furthermore, evidence from humans and rodents studies suggest that 5‐HT levels are dependent on stimulation through external social interactions (Garattini et al., 1967) and higher levels are suggested to promote pro‐social behavior (Crockett et al., 2013; Tse & Bond, 2002)—pinpointing its role in social adaptation and learning. Therefore, a focus on training communication skills and every day social interactions could have a positive reinforcing effect supporting long‐term efficacy of treatment. Therapist–patient relationship could be a starting point to re‐regulate social information processing and train social interactions. LSD has been shown to increase suggestibility to similar opinions (Duerler et al., 2020), pointing toward the importance of patient–therapist relationship quality for the potential clinical efficacy.

However, increased suggestibility as well as increased sensitivity to external social cues can have both, positive or negative consequences for the individual depending on the therapeutic environment. Therefore, therapeutical administration of psychedelics requires high responsibility and professionality and also awareness of potential risks. Supervision by professionally trained therapists with awareness and understanding of the psychedelic‐induced state, understanding of expectations of both patient and therapists, empathy with and acceptance of the patient are all critical for beneficial outcomes. Without proper adherence to these standards, the clinical administration of psychedelics may even have harmful effects. In sum, psychedelic‐assisted therapy consists of an interaction between pharmacological and non‐pharmacological/social mechanisms (Mertens & Preller, 2021). Therefore, with growing amount of research and clinical application, awareness about risks and training guidelines for therapists need to be developed to prevent potential harmful applications of these molecules.

## 8 CONCLUSION

This review summarized novel insights on the conceptual and neurobiological underpinnings of social influence processing and highlights a fundamental role of the 5‐HT system (Collins, 2001; Joiner et al., 2017; Raghanti et al., 2018). Furthermore, we discuss important implications for psychedelic‐assisted therapy.

A growing amount of research points toward 5‐HT‐mediated neuroplasticity that leads to greater adaptation by modulating processes of information integration such as increasing sensitivity to certain social cues (Branchi, 2011; Roberts et al., 2020) and regulating affective and cognitive biases of representations of context (Roberts et al., 2020; Rogers, 2011). These processes promote social learning and adaptation. Findings from neuroimaging studies reveal that processes of adaptation implicate frontal brain regions and brain networks related to the computation of value (Campbell‐Meiklejohn et al., 2010; Falk & Scholz, 2018; Klucharev et al., 2009; Wu et al., 2016). This is consistent with more recent findings from neuropharmacology and psychedelic research that demonstrate that the 5‐HT_2A_ plays a key role in the process of social information integration via modulation of the mPFC (Ambrase et al., 2020; Duerler et al., 2020; Liu et al., 2014). 5‐HT is therefore suggested to modulate integration of context‐dependent learning signals by altering self‐relevance processing that in turn affects assignment of value. This value reflects expected reward dependent on self‐relevance during value‐based decision‐making that guides learning and choice of behavior (D'Argembeau, 2013; Duerler et al., 2020; Lieberman, 2007; Roberts et al., 2020).

Therefore, these findings highlight a critical role of 5‐HT in social influence processing as it moves behavior toward greater adaptation via the modulation of social information integration as it is involved in the process of forming and updating neural representations of norms (as well as other representations such as self and other that are all interrelated in processes of alignment) (Collins, 2001; Duranton & Gaunet, 2016; Tajadura‐Jiménez et al., 2012; Tsakiris 2017). 5‐HT is therefore crucial for social learning and value‐based decision‐making—thus social adaptation

Investigating the role of the 5‐HT_2A_ receptor via the administration of serotonergic psychedelics not only revealed valuable insights for a more differentiated understanding of social influence processing, but further demonstrated how central processes of social adaptation are interrelated, such as multisensory information integration with the forming of a coherent self‐experience and its association with other‐perception during social interactions (Halberstadt & Geyer, 2013; Preller, Schilbach *et al*. 2018; Vollenweider et al., 1998). A deeper understanding on the role of the 5‐HT system in social adaptation has crucial implications for novel treatment programs. Synergistically combining pharmacological and non‐pharmacological treatments and thereby fostering novel forms of information integration may help to restructure maladaptive constraints of representations that hinder social learning and adaptive decision‐making. As 5‐HT signaling is highly intertwined with the social environment and our need for belongingness (Branchi, 2011), promoting real‐life social interactions should also be included in therapy as this could have an additional reinforcing effect and boost treatment efficacy.

In the future, leveraging modern research and analysis methods (such as computational accounts, neuroimaging techniques, real‐world social behavior beyond the laboratory) and integrating findings from different fields to better understand neurobiological mechanisms involved in distinct components of social influence will provide further insights and create a more comprehensive understanding. So far research on social influence mainly focused on mechanisms within single brains at the individual level. However, to better understand the social influence and its underlying neurobiological mechanisms, analysis of natural real‐life interactions between many brains should also be considered as our brain is embedded in a complex system of social structures. A better understanding of the neurobiology of social influence processing is not only of high relevance for psychiatric treatment but has implications for almost all parts of our daily lives.

## CONFLICT OF INTEREST

KHP is currently an employee of Hoffmann‐La Roche. The authors declare no further potential conflict of interest.

## AUTHOR CONTRIBUTIONS

PD and KHP have written the manuscript. FXV has revised the manuscript.

## Data Availability

Data sharing not applicable ‐ no new data generated, or the article describes entirely theoretical research
